# Impact of COVID-19 pandemic lockdown on myocardial infarction care

**DOI:** 10.1007/s10654-021-00764-2

**Published:** 2021-06-06

**Authors:** Timo Schmitz, Christa Meisinger, Inge Kirchberger, Christian Thilo, Ute Amann, Sebastian E. Baumeister, Jakob Linseisen

**Affiliations:** 1 Chair of Epidemiology, University of Augsburg, University Hospital Augsburg, Augsburg, Germany; 2 Independent Research Group Clinical Epidemiology, German Research Center for Environmental Health, Helmholtz Zentrum München, Munich, Germany; 3 Department of Internal Medicine I - Cardiology, University Hospital of Augsburg, Augsburg, Germany; 4 University Hospital Münster, Münster, Germany

**Keywords:** Covid-19, Myocardial infarction, Lockdown, Door-to-device-time, Population-based registry, Augsburg, Bavaria, Germany

## Abstract

The aim of this study was to evaluate the impact of the COVID-19 pandemic lockdown on acute myocardial infarction (AMI) care, and to identify underlying stressors in the German model region for complete AMI registration. The analysis was based on data from the population-based KORA Myocardial Infarction Registry located in the region of Augsburg, Germany. All cases of AMI (*n* = 210) admitted to one of four hospitals in the city of Augsburg or the county of Augsburg from February 10th, 2020, to May 19, 2020, were included. Patients were divided into three groups, namely pre-lockdown, strict lockdown, and attenuated lockdown period. An additional survey was conducted asking the patients for stress and fears in the 4 weeks prior to their AMI. The AMI rate declined by 44% in the strict lockdown period; in the attenuated lockdown period the rate was 17% lower compared to the pre-lockdown period. The downward trend in AMI rates during lockdown was seen in STEMI and NSTEMI patients, and independent of sex and age. The door-to-device time decreased by 70–80% in the lockdown-periods. In the time prior to the infarction, patients felt stressed mainly due to fear of infection with Sars-CoV-2 and less because of the restrictions and consequences of the lockdown. A strict lockdown due to the Covid-19 pandemic had a marked impact on AMI care even in a non-hot-spot region with relatively few cases of COVID-19. Fear of infection with the virus is presumably the main reason for the drop in hospitalizations due to AMI.

## Introduction

Since the first reported case of Covid-19 on January 27, 2020, in Bavaria, [1] Germany, the number of confirmed cases increased rapidly in early March. The exponential increase in newly confirmed cases reached a total of 67,366 positively tested cases on April 1 in whole Germany [2] and 20,178 cases in Bavaria, the German federal state most affected by Covid-19. Within Bavaria, counties were affected to varying degrees by the pandemic; the city of Augsburg and the district of Augsburg were among the less affected counties (on April 1, 2020: 454 Covid-19-cases) [2]. On March 16, the Bavarian Government declared a state of emergency and enforced non-pharmaceutical interventions. These included physical distancing, hygiene, masks, isolation of infected people and their contacts, and lockdowns, such as closures of schools and businesses and bans on public gatherings and travel. To preserve resources, the Bavarian Government recommended deferral of elective procedures in patient care but not of care for emergency conditions such as acute myocardial infarction (AMI). Several recent studies, which were conducted in Covid-19 epicenters, reported significant reductions of hospitalizations due to AMI during the onset of the Covid-19 pandemic.[3–7] So far, investigations of heart attack events in a region with relatively low numbers of Covid-19 cases but an early and strict lockdown are missing. Because the Augsburg area is one of the districts with fewer Covid-19 cases, we evaluated AMI care before, during and post the lockdown using data from the well-established, population-based KORA Myocardial Infarction Registry in Augsburg, [8] Germany, covering all AMI events in a region of about 680,000 inhabitants. In addition, we examined possible stressors that prevented acute heart attack patients from seeking immediate inpatient treatment.

## Methods

### Study population

The underlying data for this analysis was collected prospectively by the KORA Myocardial Infarction Registry. The data collection of the registry is population-based with consecutive enrollment of all cases of non-fatal AMI within the study region. The inclusion criteria are the following: the patient is older than 24 years, survived more than 24h in the hospital, and is an inhabitant of the study region of Augsburg (city of Augsburg and two adjacent counties Aichach-Friedberg and Augsburg). Detailed information on case identification and data collection is given elsewhere. [8, 9] For the present study, all cases of AMI admitted from February 10, 2020, to May 21, 2020, were included. Patients were divided into three groups: pre-lockdown (February 10th to March 15th, 2020), strict lockdown (March 16th to April 19th, 2020), and attenuated lockdown (April 20th to May 21th) period. Only patients treated in the University hospital of Augsburg and 3 other hospitals located in the city of Augsburg and the county Augsburg were considered (about 550,000 inhabitants). Of those, 2 hospitals (in particular the University hospital of Augsburg) perform cardiac catheterization; the 2 hospitals without cardiac catheterization laboratories transfer the patients to the University hospital of Augsburg for invasive treatment of AMI. A total of 210 at least 24 h surviving AMI cases were treated in one of the 4 hospitals in this region within the study period. For the survey study, a questionnaire was sent out to 90 patients, of which 61 replied instantly or after a postal reminder (67.8%). Another 58 patients completed the questionnaire as a part of the routine interview (total response: 119). The study complies with the Declaration of Helsinki. All study participants gave written informed consent and the study was approved by the Ethics Committee of the Bavarian Medical Association (Bayerische Landesärztekammer).

### Data collection

The study participants were interviewed shortly after intensive care by study nurses and further data were collected by review of the medical chart and discharge report. Information on the acute event (time of symptom onset), treatment procedures (PCI, coronary artery bypass grafting) complications during hospital stay (cardiogenic shock, ventricular fibrillation, in-hospital mortality), cardiopulmonary resuscitation in- or outside the hospital, type of AMI (STEMI/NSTEMI), time from symptom onset to hospital admission, time from admission to revascularization, and information on physician diagnosis of diabetes were gathered.

In addition to the routinely collected data, an observational, cross-sectional study on perceived stress was performed. A questionnaire was created, which contained two single items on a past or current infection with SARS-CoV-2 among the respondents and infections in their social environment. Further, the intensity of perceived stress with regards to 19 different situations, events or feelings in the last 4 weeks before the MI was requested. The question was: “In the 4 weeks before your heart attack, how much did you feel stressed by the following situations, events or feelings?”. The response options were: 1 = not at all/does not apply, 2 = slightly, 3 = moderately, 4 = severely, 5 = very severely. For this survey, only patients who were included in the routine data collection of the registry were considered. For patients with AMI between early March and mid-May the questionnaires were sent out on May, 18th and in case of non-responding, a postal reminder was sent out on June, 10th. Patients with AMI between mid-May and end of June received the questionnaire in the context of the interview that was conducted routinely as part of the data collection of the registry. AMI patients who died during their hospital stay were not considered for the survey.

**Fig. 1:**
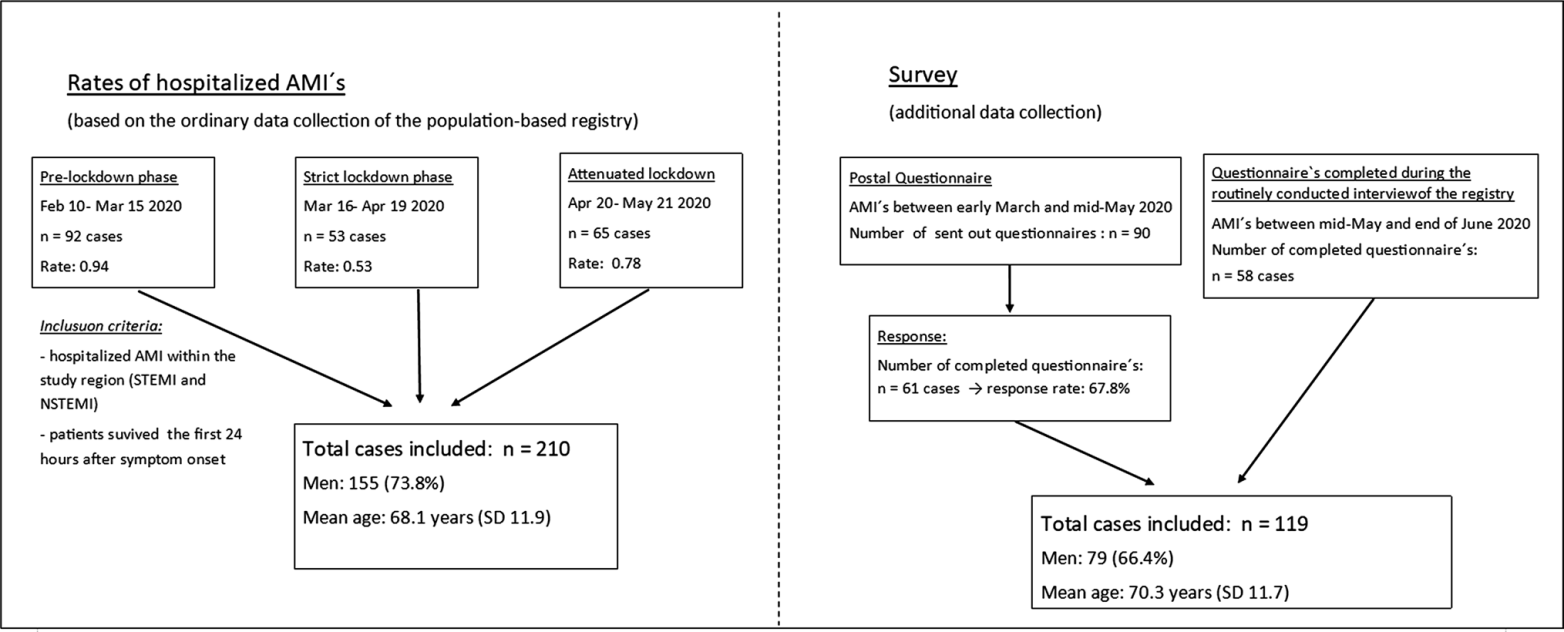
A flow chart displaying the study population and the process of data collection for the rates of hospitalized AMI´s and for the survey

Figure 1 provides a Flow Chart, which illustrates the process of data collection for the hospitalized AMI´s and the additional survey.

### Statistical analysis

Summary statistics were provided for the pre-lockdown period and two the lockdown periods. We calculated AMI rates with exact Poisson 95% confidence intervals (CI). Cumulative person time (in years) since the beginning of the study period (i.e., February 10th, March 16th, and April 20th) was computed. Age- and sex-adjusted rate ratios (RR) and time ratios (TR) for the comparison of the two lockdown periods and the pre-lockdown period were estimates using negative binomial regression models.

For the multi-item questionnaire on perceived stress, a factor analysis was conducted to clarify whether a summary score can be calculated. Adequacy of the data for factor analysis was assessed by performing Bartlett-Test for significant correlation of the items and calculating values of the Kaiser–Meyer–Olkin-Test (KMO). The appropriate number of factors was determined according Valicer’s minimum average partial test and parallel analysis. Finally, principal component analysis with varimax rotation was performed. Differences between the factors were analyzed by performing analysis of variance (ANOVA) and post-hoc-t-tests with Bonferroni adjustment. *P*-values < 0.05 were considered statistically significant. All analyses were performed using R (version 3.6.3).

## Results

A total of 210 patients with AMI were included in the analysis of infarction rates before and during the first lockdown in Germany. Table 1 depicts patient characteristics according to one pre-lockdown period and two lockdown periods. About 70% of the study population were men. The mean age was 68.1 (standard deviation [SD]: 11.9) in men and 73.9 (SD: 13.5) in women. Overall, 42.4% were diagnosed with a STEMI, 55.7% had an NSTEMI, 31.0% had a diagnosis of type 2 diabetes, 78.6% had their first AMI, 6.2% died during the hospital stay, and 10.5% showed in-hospital complications.

**Table 1:** Patient characteristics (n (%)) before and during two lockdown periods due to the Covid-19 pandemic

	Pre-lockdown phase (*n* = 92) Feb 10–Mar 15 2020	Strict lockdown phase (*n* = 53) Mar 16–Apr 19 2020	Attenuated lockdown phase (*n* = 65) Apr 20–May 21 2020
Men	67 (72.8)	40 (75.5)	39 (60.0)
*Age*			
< 55 years	13 (14.6)	5 ( 9.6)	6 ( 9.8)
55–65 years	24 (27.0)	16 (30.8)	15 (24.6)
66–74 year	12 (13.5)	13 (25.0)	13 (21.3)
75 + years	40 (44.9)	18 (34.6)	27 (44.3)
STEMI	39 (42.4)	24 (45.3)	26 (40.0)
NSTEMI	51 (55.4)	29 (54.7)	37 (56.9)
Diabetes	31 (33.7)	14 (26.4)	20 (30.8)
First infarction	76 (82.6)	38 (71.7)	51 (78.5)
In-hospital mortality	6 ( 6.5)	1 ( 1.9)	6 ( 9.2)
Complication	9 ( 9.8)	3 ( 5.7)	10 (15.4)

STEMI: ST-segment elevation myocardial infarction. NSTEMI: non-ST-segment elevation myocardial infarction. Complication includes out-of-hospital or in-hospital resuscitation, in-hospital cardiogenic shock, and in-hospital ventricular fibrillation

The AMI rate declined by 44% (95% CI: 53%-34%) in the first period after lockdown compared to the pre-lockdown phase (Table 2). During the second post-lockdown period, the rate was 17% lower compared to the pre-lockdown period (RR = 0.83, 95% CI 0.70–0.96). The downward trend in myocardial infarction rates during lockdown was similar in men and women, and STEMI and NSTEMI patients. In comparison to the pre-lockdown period, the decline in myocardial infarction rates in the strict lockdown period was most pronounced in patients aged 55 or less (RR = 0.29, 95% CI 0.15–0.42), those aged 66–74 years (RR = 0.26, 95% CI 0.16–0.37), and individuals aged 75 or more (RR = 0.28, 95% CI 0.21–0.35). In the age group 55–65 years there was a decline in infarction rates (RR = 0.41, 95% CI 0.29–0.54) as well, yet less pronounced than in other age groups. During the attenuated lockdown phase, rates tended to move back to pre-lockdown levels.

**Table 2:** Rate ratios and time ratios for hospital admission of patients with acute myocardial infarction, symptom-to-door and door-to-device time before and during the two lockdown periods due to the Covid-19 pandemic

	Pre-lockdown	Strict lockdown	Attenuated lockdown
	Feb10- Mar 15	Mar 16- Apr 19	Apr 20- May 19
*Total sample*			
Rate *(95% CI)*	0.94 (0.02; 5.44)	0.53 (0.01; 2.96)	0.78 (0.02; 4.33)
Rate ratio (95% CI)	1.0	0.56 (0.47; 0.66)	0.83 (0.70; 0.96)
*Men*			
Rate *(95% CI)*	1.72 (0.04; 9.6)	0.92 (0.02; 5.11)	0.98 (0.02; 5.47)
Rate ratio (95% CI)	1.0	0.53 (0.36; 0.70)	0.57 (0.41; 0.73)
*Women*			
Rate *(95% CI)*	0.68 (0.02; 3.79)	0.31 (0.01; 1.71)	0.46 (0.01; 1.71)
Rate ratio (95% CI)	1.0	0.46 (0.37; 0.54)	0.68 (0.55; 0.81)
*Age****:*** < *55 years*			
Rate *(95% CI)*	0.94 (0.02; 5.26)	0.27 (0.01; 1.52)	0.49 (0.01; 2.73)
Rate ratio (95% CI)	1.0	0.29 (0.15; 0.42)	0.52 (0.29; 0.76)
*Age: 55–65 years*			
Rate (95% CI)	1.21 (0.03; 6.67	0.50 (0.01; 2.77)	1.03 (0.03; 5.75)
Rate ratio (95% CI)	1.0	0.41 (0.29; 0.54)	0.85 (0.58; 1.12)
*Age: 66–74 years*			
Rate (95% CI)	2.09 (0.05; 11.63)	0.55 (0.01; 3.07)	1.57 (0.04; 8.77)
Rate ratio (95% CI)	1.0	0.26 (0.16; 0.37)	0.75 (0.46; 1.05)
*Age: 75* + *years*			
Rate (95% CI)	4.62 (0.12; 25.76)	1.29 (0.03; 7.17)	1.48 (0.04; 8.24)
Rate ratio (95% CI)	1.0	0.28 (0.21; 0.35)	0.32 (0.24; 0.40)
*STEMI*			
Rate *(95% CI)*	0.84 (0.01; 5.24)	0.42 (0.01; 2.36)	0.68 (0.02; 3.78)
Rate ratio (95% CI)	1.0	0.50 (0.38; 0.62)	0.81 (0.61; 1.01)
*NSTEMI*			
Rate (95% CI)	0.36 (0.01, 2.02)	0.23 (0.01; 1.28)	0.40 (0.01; 2.24)
Rate ratio (95% CI)	1.0	0.64 (0.50; 0.78)	1.11 (0.88; 1.34)
**Symptom-to-door time** *Total sample*			
Hours (mean, sd)	21.2 (49.5)	67.1 (355.8)	3.3 (3.7)
Time ratio (95% CI)	1.0	4.25 (2.13; 8.82)	0.18 (0.09; 0.33)
*STEMI*			
Hours (mean, sd)	10.5 (24.9)	3.3 (4.3)	2.9 (3.0)
Time ratio (95% CI)	1.0	0.31 (0.15; 0.69)	0.30 (0.15; 0.63)
*NSTEMI*			
Hours (mean, sd)	31.1 (62.9)	138.9 (517.7)	3.7 (4.2)
Time ratio (95% CI)	1.0	6.68 (2.51; 19.50)	0.13 (0.06; 0.31)
**Door-to-device time** *Total sample*			
Hours (mean, sd)	30.2 (104.4)	5.2 (6.2)	7.0 (9.2)
Time ratio (95% CI)	1.0	0.23 (0.13; 0.42)	0.26 (0.15; 0.44))
*STEMI*			
Hours (mean, sd)	3.6 (6.5)	1.0 (0.6)	1.4 (1.5)
Time ratio (95% CI)	1.0	0.26 (0.14; 0.49)	0.35 (0.19; 0.63)
*NSTEMI*			
Hours (mean, sd)	52.3 (146.6)	10.9 (5.7)	12.1 (10.2)
Time ratio (95% CI)	1.0	0.19 (0.09; 0.40)	0.20 (0.11; 0.37)

Rate per cumulative person time (in years). CI denotes confidence interval, STEMI: ST-segment elevation myocardial infarction and NSTEMI: non-ST-segment elevation myocardial infarction

Overall, the time ratio of symptom-to-door time increased from 1.0 (reference) in the pre-lockdown phase to 4.25 (95% CI 2.13–8.82) in the strict lockdown phase (which is equivalent to an increase of 325% (95% CI 113%-782%)). Thereafter, time ratio of symptom-to-door time decreased to 0.18 (95% CI 0.09–0.33) for the attenuated lockdown phase. However, there were notable differences between cases diagnosed with STEMIs and NSTEMIs. The symptom-to-door times shortened in STEMI (TR = 0.31, 95% CI 0.15–0.69) and increased in NSTEMI (TR = 6.68, 95% CI 2.51–19.50) cases. The door-to-device time decreased 70–80% during lockdown-periods compared to the pre-lockdown phase, overall, and in the STEMI and NSTEMI cases.

### Survey

A total of 119 patients completed the questionnaire. Of those, 61 answered to the postal questionnaire (out of 90 who received the questionnaire, response rate: 67.8%). Another 58 questionnaires were carried out in the hospital as part of the routinely conducted interview. Mean age of the patients included in the survey was 70.3 years (SD: 11.7); 79 of those were men (66.4%). A total of 113 (95.0%) patients had no infection with Sars-CoV-2 until the time of the interview. One patient (0.8%) indicated a current infection and one patient (0.8%) had already recovered from Covid-19. Another 4 patients (3.4%) stated they don´t know whether they had an infection with Sars-CoV-2. Most patients (*n* = 99, 83.2%) had no affected persons in their social environment. There were no confirmed current infections among the patients’ social environment, but 3 patients (2.5%) claimed that there are unconfirmed suspected cases. Additionally, 5 patients (4.2%) stated that there are recovered persons, and 3 patients (2.5%) had persons in their social environment, who died from Covid-19.

The main question on perceived stress (19 items) aimed at situations, occasions, and feeling, by which patients felt stressed or burdened during the 4 weeks before AMI. Detailed results are displayed in Table 3. The factor analysis revealed an optimum of 4 factors (see Tables 3, 4), which are the following: *Factor 1*: job/finances (5 items). *Factor 2*: family/friends/social environment (7 items). *Factor 3*: infection with coronavirus (3 items). *Factor 4*: society/social life (3 items). Based on these results, corresponding subscale scores were built by adding the responses of the single items.

**Table 3:** Perception of stress in patients with acute myocardial infarction: Answers to the question: “In the 4 weeks before your heart attack, how much did *you feel stressed by the following situations, events or feelings?*” are displayed as total number (percentages) and mean (SD)

	Factor	*n*	Not at all/does not apply	Slightly	Moderately	Severely	Very severely	Mean (SD)
Conflicts with related persons	2	117	84 (71.8%)	18 (15.4%)	8 (6.8%)	5 (4.3%)	2 (1.7%)	1.49 (0.92)
Dependents in need of care	2	116	100 (86.2%)	8 (6.9%)	4 (3.4%)	1 (0.9%)	3 (2.6%)	1.27 (0.80)
Constant accessibility	2	117	81 (69.2%)	17 (14.5%)	8 (6.8%)	6 (5.1%)	5 (4.3%)	1.61 (1.10)
Many private obligations	2	116	81 (69.8%)	20 (17.2%)	5 (4.3%)	7 (6.0%)	3 (2.6%)	1.54 (1.01)
Death of related person	2	116	105 (89.7%)	5 (4.3%)	2 (1.7%)	2 (1.7%)	3 (2.6%)	1.23 (0.79)
Worries about health of a related person	2	118	61 (51.7%)	26 (22.0%)	14 (11.9%)	10 (8.5%)	7 (5.9%)	1.95 (1.23)
Separation from partner	2	118	109 (92.4%)	3 (2.5%)	0 (0.0%)	2 (1.7%)	4 (3.4%)	1.21 (0.83)
Current financial situation	1	117	88 (75.2%)	17 (14.5%)	7 (6.0%)	3 (2.6%)	2 (1.7%)	1.41 (0.85)
Financial future	1	117	81 (69.2%)	24 (20.5%)	8 (6.8%)	2 (1.7%)	2 (1.7%)	1.46 (0.84)
Current job-related situation	1	116	89 (76.7%)	8 (6.9%)	9 (7.8%)	5 (4.3%)	5 (4.3%)	1.53 (1.09)
Future job-related situation	1	116	93 (80.2%)	8 (6.9%)	7 (6.0%)	2 (1.7%)	6 (5.2%)	1.45 (1.05)
Loneliness/isolation	4	118	85 (72.0%)	18 (15.3%)	10 (8.5%)	3 (2.5%)	2 (1.7%)	1.47 (0.88)
Limited leisure activities	4	117	65 (55.6%)	17 (14.5%)	16 (13.7%)	14 (12.0%)	5 (4.3%)	1.95 (1.25)
Family situation	2	117	80 (68.4%)	20 (17.1%)	7 (6.0%)	9 (7.7%)	1 (0.9%)	1.56 (0.97)
Feeling of excessive demands	1	117	82 (70.1%)	15 (12.8%)	8 (6.8%)	8 (6.8%)	4 (3.4%)	1.61 (1.1)
Less contact with other people	4	118	68 (57.6%)	22 (18.6%)	15 (12.7%)	10 (8.5%)	3 (2.5%)	1.85 (1.11)
Health situation in Germany	3	113	54 (47.8%)	38 (24.8%)	16 (14.2%)	12 (10.6%)	3 (2.7%)	1.96 (1.14)
Fear of infection with new Corona-virus	3	118	51 (43.2%)	43 (36.4%)	13 (11.0%)	5 (4.2%)	6 (5.1%)	1.92 (1.08)
Fear of infection of a related person with new Corona-virus	3	117	40 (34.2%)	42 (35.9%)	17 (14.5%)	12 (10.3%)	6 (5.1%)	2.16 (1.16)

**Table 4:** A factor analysis was conducted to characterize perception of stress in patients with acute myocardial infarction

Actor analysis	ANOVA *p*-value: < 0.0001	Post-hoc-t-test (Bonferroni adjustment) *p*-values		
	mean (SD)	Factor 2	Factor 3	Factor 4
Factor 1: Job/finances	1.49 (0.99)	1	< 0.0001	0.00253
Factor 2: Family/friends/social environment	1.48 (0.99)	–	< 0.0001	0.00049
Factor 3: Fear of infection with Sars-Cov-2	2.01 (1.13)	–	–	0.00260
Factor 4: Society/social life	1.74 (1.11)	–	–	–

The factor analysis revealed an optimum of 4 factors, which are the following: *Factor 1:* job/finances; *Factor 2:* family/friends/social environment; *Factor 3:* fear of infection with Sars-Cov-2; *Factor 4:* society/social life. An analysis of variance (ANOVA) revealed significant differences among the factors. Post-hoc t-tests were performed to further analyze differences between each of the 4 factors. Factor 3 had significant higher values than the remaining factors. Factor 4 was rated significantly higher than factor 2 and 1. The two latter ones did no vary significantly from each other

**Fig. 2:**
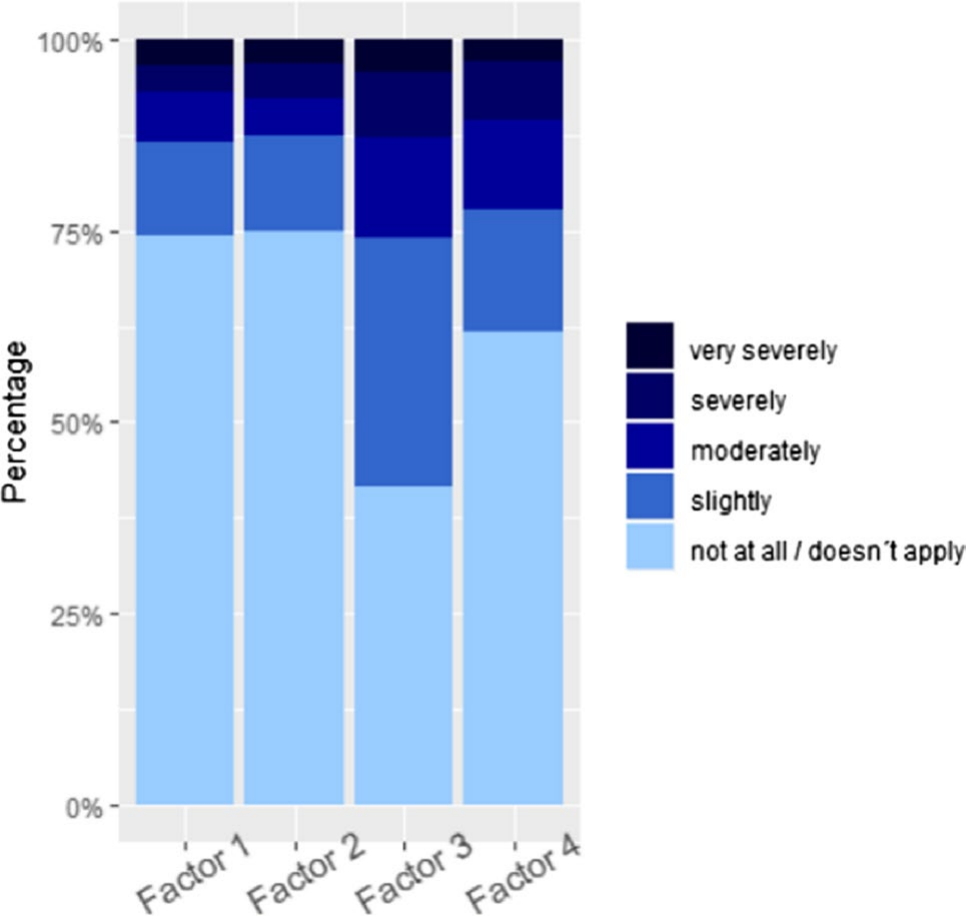
The main question of the survey was: “In the 4 weeks before your heart attack, how much did you feel stressed by the following situations, events or feelings?” (see Table 3 for the all items of this question). Figure 2 displays the percentages of given answers for each of the 4 factors that were identified by factor analysis (see Table 4): *Factor 1: stress by job/finances, Factor 2: stress due to family/friends/social environment, Factor 3: stress caused by fear of infection with Sars-Cov-2, Factor 4: stressed by society/social life. Figure generated with* R version 3.6.3

Analysis of variance revealed significant differences between the factors. Post-hoc t-tests using Bonferroni adjustment were performed to further analyze the differences (see Table 4 and Fig. 2). It was found, that stress due to fear of infection with Sars-CoV-2 (factor 3) was significantly more frequent among the patients than stress due to the three other categories. Stress caused by the restrictions in social life/society (factor 4: loneliness/isolation, limited social contact and leisure activities) was significantly higher than stress generated by job/finances (factor 1) and family/friends/social environment (factor 2).

## Discussion

We observed a reduction in AMI cases by almost 50% in the five weeks of strict lockdown in our non-hot-spot Covid-19 study region. A reduction was seen in STEMIs as well as NSTEMI cases, the latter with an even greater drop in numbers. Men and women and age groups were similarly affected by the downturn. In addition, our study showed a reduction in door-to-device time for both patients with STEMI and NSTEMI. In contrast, the symptom-to-door-time decreased in STEMI patients but increased markedly in NSTEMI patients during the strict lockdown. During the attenuated lockdown phase, a trend towards the conditions of the pre-lockdown period is seen. It seems that the predominant reason for the drop of cases was stress by fear of infection with the virus.

Although our study area was not located in an epicenter of the pandemic in spring 2020, there has been a massive decrease in AMI cases during the lockdown period, a finding almost similar to reports from Austria, [7] Italy, [4, 5] North America, [3, 6, 10–12] and Spain, [13] in which mostly data from pandemic epicenters were analyzed. In this study, the door-to-device time for both STEMI and NSTEMI patients diminished during the lockdown. On the other hand, there was an increase in symptom-to-door time among NSTEMI patients. An early study from Hong Kong, [14] China, suggested a large delay in the time from symptom onset to first medical contact after the onset of the pandemic. Contrary to our findings, an investigation from another non-hot-spot region in the US reported a delay in presentation and a longer average door-to-device time in STEMI patients. The authors suggested that these delays were due to fear of Covid-19, or in belief of patients that the symptoms were Covid-19 related, or that patients did not want to burden the emergency department during the Covid-19 pandemic. [11] The pre-hospital phase of ischemic duration, particularly in STEMI, is strongly associated with patient outcomes, [15, 16] and the in-hospital timing of treatment is critically important. [17, 18] Short door-to-device-times are key components of efficient reperfusion therapy in AMI care [19].

In contrast to the present findings, one could have expected a rise in AMI cases due to various reasons. Psychosocial and environmental stress caused by the pandemic might have led to an increased incidence of AMI. Moreover, higher numbers of infarction cases could be induced by respiratory infections and viral illness due to 2019 novel Coronavirus (SARS-CoV-2), which can also be observed for influenza infections [20].

There are several possible explanations for the drop in cases. One major hypothesis is, that the reduction in hospitalizations due to AMI is caused by an actual reduction in cases, which could be a result of reduced physical stress and working stress during quarantine and lockdown times. However, the decrease in cases can be observed in all age groups including patients aged 70 years or older. Since most people in this age group are retired, the argument of reduction of physical stress or working stress is probably not valid. Moreover, meta-analyses concluded, that the pandemic has also caused additional psychological stress, fears and anxiety as well as a number of newly diagnosed mental disorders [21, 22]. Consequently, it appears implausible, that reduced levels of stress as a consequence of the lockdown measures led to lower numbers of AMI in March and April.

The second main hypothesis is a reduced willingness to seek medical help by the patients. The main reason for this might be fear of in-hospital infection with SARS-CoV-2 and a lower perceived necessity to seek help. This effect probably applies primarily to milder events. The results of our patient survey via questionnaire revealed, that in the 4 weeks prior to the event, patients were mainly stressed by fear of infection with the virus (infection of themselves and even more by fear of infections of close relatives) and by the health situation in Germany itself. This strengthens the hypothesis that patients with typical AMI symptoms hesitated to seek medical help due to fear of infection in the hospital. We assume this to be the predominant reason for the drop of cases in the early weeks of the lockdown. Ciofani et al. reported, that the internet search volume for chest pain strongly correlated with Covid-19 case numbers in the USA [23]. They suspected, that fear of Covid-19 may lead patients to self-triage using internet research and in this way avoid hospital visits, which would be in agreement with our results.

Our survey further revealed, that situations and events other than fear of Covid-19 generated less stress. Restrictions in social life (reduced contact to others, loneliness/isolation, limited possibilities of free time activities) caused a medium amount of stress in the 4 weeks prior to the event. These restrictions are mostly caused by lockdown and containment measures. Nevertheless, they generated less stress and worries than the virus itself.

The least amount of stress was caused by affaires concerning family, friends or close social environment (e.g. conflicts, private obligations or separation from partner) and financial and job-related worries. The latter might be explained on the one hand by the presumabley large number of patients who are already retired and not dependent on jobs for their financial incomes. On the other hand, this survey was conducted in an early phase of the pandemics (March to June) and economic consequences like job losses and insolvencies of companies emerged mainly in the further course of the year 2020.

Overall, fear of infection and the health situation in Germany was by far the most important source of stress. Furthermore, patients felt stressed more through restrictions of social life and less through financial or job-related worries and affairs of close social environment. It has to be considered, that persons experiencing a heart attack do not represent the general population as they are older and have more comorbidities and are predominately men. So, these results are specifically valid for patients with a high risk of AMI and not for other groups of patients. Additionally, it has to be mentioned, that the patients who presumably had a MI but did not seek medical help are missing in this survey. It remains unclear, if their fears and worries differ from those of the patients who participated in this survey.

As a consequence of the fear of Covid-19 disease and the hesitation to seek medical help, it wouldn´t be surprising to see an increase in prehospital time. This is what other researchers have found after onset of the corona pandemics in other countries [11, 14]. Nevertheless, we observed this in our data for NSTEMI patients only. In fact, there was a drop in prehospital time for STEMI patients and a decrease in door-to-device time in both STEMI and NSTEMI patients compared to the time-period before the lockdown. This reduction in symptom-to-door time for STEMI and door-to-device-time might, in part, be explained by a higher proportion of more severe cases, which are usually characterized by more pronounced symptoms.

Another fact that needs to be considered when interpreting the data is that the Covid-19 pandemic could be attenuated at an early phase in spring 2020 in Germany, except for a few cluster events (e.g. the district of Heinsberg), and the health care system was never overwhelmed during the outbreak. This also applies to the hospitals in the study region. As in non-Covid-19 times, the PCI resources were kept running during the pandemic. Thus, a short door-to-device time for the STEMIs and NSTEMIs could be maintained throughout, and even improved overall. A likely explanation is that the treatment of elective cases was eliminated during the lockdown, and therefore sufficient treatment capacities were available at all times for AMI patients. This difference in comparison to other, harder-hit countries might be the main explanation for the reduction in door-to-device time.

Untreated AMI can cause severe complications and substantial vascular damage, including heart failure, [24] ventricular arrhythmias, [25] ventricular remodeling [26] and premature death. As our data can be considered representative due to the population-based structure of this registry, a transfer of our findings to the whole country results in a considerable reduction of medically treated AMI cases for the calendar weeks 12 to 16. Assuming most of missing cases still occurred during this time but simply without adequate treatment, this would mean substantial collateral damage. Fortunately, the quality of medical care for patients with AMI seeking for help remained on the same high level during the lockdown as it was before the pandemic. In this regard, it seems to be important to encourage people to seek help when they experience chest pain in times of a pandemic.

The present study has some limitations and strengths. Data collection during a pandemic is challenging because it is necessary to provide human resources for the care of Covid-19 patients. Thus, extensive data collection by a detailed chart review was not possible. Therefore, some relevant information such as behavioral risk factors, comorbidities, and laboratory parameters were unavailable for the present study.

Further to mention is a relatively small sample size of 53 recorded cases of hospitalized myocardial infarction during the strict lockdown phase and a total of 119 patients that completed the questionnaire. Patients that participated in the survey were recruited in two different ways: patients with AMI in the early phase of the lockdown received a postal invitation and patients with AMI in the attenuated lockdown phase completed the questionnaire during their hospital stay as part of the common data collection of this registry. This circumstance might have led to a selection, recall or interviewer bias.

Nonetheless, the questionnaire on perceived stress of the patients in the time 4 week prior to the events gives additional insights and draws a larger picture of the situation. The questionnaire was self-developed and specifically designed for the pandemic situation in order to address the uniqueness of the situation as best as possible. An evaluation of its psychometric properties was not possible prior to the use in the current study, which must be considered as weakness.

A major strength of this study is the use of the population-based registry and its consecutive enrollment of patients within a defined study region including urban and rural areas. This excludes relevant selection bias and ensures that results from this registry are fairly representative for the German population. Since the KORA Myocardial Infarction Registry prospectively collects all cases of AMI in the study region of Augsburg, subsequent investigations on the effects of the lockdown phase on fatal (including pre-hospital deaths) and non-fatal events and on the occurrence of re-infarctions and complications can further contribute to the understanding of those issues.

## Data Availability

The data underlying this article cannot be shared publicly because the data are subject to national data protection laws and restrictions that were imposed by the ethics committee of the Bavarian Medical Association ("Bayerische Landesärztekammer") to ensure data privacy of the study participants because they did not explicitly consent to the data being madepublicly available. The data will be shared on reasonable request to the corresponding author.

## Code availability

Available on reasonable request to the corresponding author.
